# Supplementary material for: Comprehensive Annotation and Functional Exploration of MicroRNAs in Lettuce
Source: Front Plant Sci. 2021 Dec 24;12:781836. doi: 10.3389/fpls.2021.781836 (PMC8739914; doi:10.3389/fpls.2021.781836)
Supplement: Supplementary file 3 [file Data_Sheet_9.ZIP › GSM2230752.plot/Lsa-miR168a_Lsat_1_v5_gn_3_35820.1_1750_TPlot.pdf]

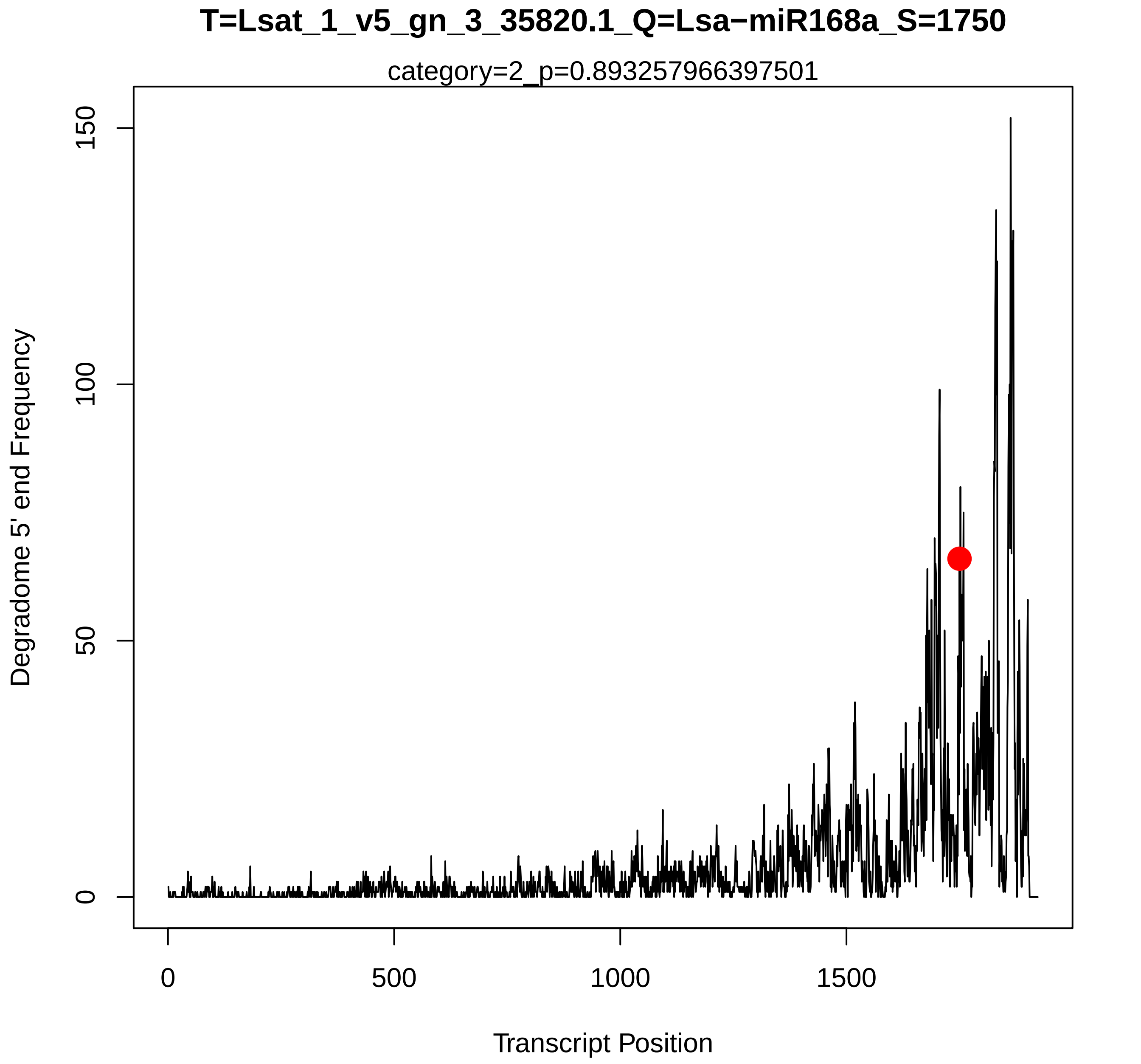

T=Lsat_1_v5_gn_3_35820.1_Q=Lsa−miR168a_S=1750
category=2_p=0.893257966397501
Degradome 5' end Frequency
150
100
50
0
0
500
1000
1500
Transcript Position
